# Respiratory viral detection and severity risk factors in hospitalized children with community-acquired pneumonia following relaxation of COVID-19 non-pharmaceutical interventions: a 20-month retrospective cohort in Guilin, China

**DOI:** 10.7717/peerj.21849

**Published:** 2026-11-09

**Authors:** Xin Wang, Junli Bu, Qiqing Wan, Zhi Yin, Xiaoying Zhang, Yiping Gu

**Affiliations:** 1 The School of Public Health, Guilin Medical University, Guilin, China; 2 The Affiliated Hospital, Guilin Medical University, Guilin, China; 3 School of Medicine, Lishui University, Lishui, China

**Keywords:** Community-acquired pneumonia, Non-pharmaceutical interventions, Retrospective cohort, Children, Risk factor, Viral pathogens

## Abstract

**Background:**

After the withdrawal of COVID-19 non-pharmaceutical interventions (NPIs) in China, we characterized the demographic, clinical and virological features of virus-positive community-acquired pneumonia (CAP) in children younger than 5 years during the post-NPI period.

**Methods:**

We conducted a retrospective study using electronic medical records from a tertiary hospital in Guilin, China. We enrolled 1,255 children hospitalized with CAP between December 2022 and July 2024. All participants were tested for five respiratory viruses: respiratory syncytial virus (RSV), parainfluenza virus (PIV), adenovirus (ADV), influenza-A (Flu-A) and influenza-B (Flu-B).

**Results:**

Of 1,255 hospitalized CAP cases, 681 (54%) were virus-positive, including 112 (16% of virus-positive cases) who met severe CAP (SCAP) criteria. RSV showed the highest detection rate (23%, 292/1,255), followed by PIV, ADV, Flu-A and Flu-B; detection patterns differed significantly across age groups. Bacterial co-detection was common among virus-positive cases. Multivariable analysis identified bacterial co-detection (OR 1.90, 95% CI [1.12–3.23]), wheeze (OR 1.59, 95% CI [1.02–2.49]), and chronic comorbidities (OR 2.44, 95% CI [1.5 7–3.78]) as independent risk factors for severe disease, whereas rhinorrhoea was associated with reduced severity (OR 0.43, 95% CI [0.27–0.66]).

**Conclusion:**

Among virus-positive cases, RSV was the most frequently detected virus in children younger than 5 years hospitalized with CAP during the post-NPI period in Guilin, China. Early identification of independent risk factors for SCAP may help clinicians’s recognition high-risk children promptly.

## Introduction

Community-acquired pneumonia (CAP) remains a significant health burden among children under 5 years of age (Pratt et al., 2022). Annually, approximately 120 million cases of CAP occur globally, resulting in approximately 1 million deaths (McAllister et al., 2019). Viral pathogens—particularly respiratory syncytial virus (RSV), influenza virus, and parainfluenza virus (PIV)—are major pathogens of CAP in young children (Dagan et al., 2023; Haynes et al., 2014; Jain, 2017; Pratt et al., 2022). Advances in diagnostic technology have underscored the important role of viruses in the etiology of CAP and substantially improved the identification of viral pathogens in affected patients (Burk et al., 2016). However, transmission routes vary by pathogen. RSV spreads mainly *via* droplets and contact; therefore, hand hygiene and surface cleaning remain crucial (Kaler et al., 2023). PIV share similar transmission routes, underscoring the need for strict surface hygiene (Kim et al., 2017). Influenza A (Flu-A) and influenza B (Flu-B) spread *via* droplets and aerosols, their short incubation period and pre-symptomatic transmission mean early antiviral treatment and contact tracing are critical (Patrozou & Mermel, 2009). Adenovirus (ADV) transmits through droplets, contact, and the fecal-oral route; it propagates rapidly in childcare facilities and homes because young children frequently touch their mouths; thus, chlorine-based disinfection is needed to stop outbreaks (Doerfler, 1996).

Accumulating evidence indicates that certain viruses—such as RSV, severe acute respiratory syndrome coronavirus (SARS-CoV), influenza virus, and ADV—are associated with the severe disease and mortality in pediatric CAP (Chen et al., 2024; Haynes et al., 2014; Jain, 2017; Pratt et al., 2022). However, the dominant viral profile fluctuates over time and across regions, as the COVID-19 pandemic and its non-pharmaceutical interventions (NPIs) have reshaped respiratory-virus epidemiology in the post-pandemic period (Eden et al., 2022; Li et al., 2025). Systematic surveillance of viral CAP is essential for identifying predominant pathogens and risk factors. Such data can guide public health resource allocation, inform immunization polices, and improve clinical management, helping to reduce the childhood burden of viral CAP in the post-pandemic era (Eden et al., 2022; Scaglione et al., 2025). Based on these considerations, the present study examined the epidemiological profile of five viruses commonly detected in pediatric CAP—RSV, ADV, PIV, influenza-A (Flu-A), and influenza-B (Flu-B)—and aimed to identify the risk factors associated with virus-positive CAP severity.

## Materials and Methods

### Study design

This retrospective cohort study was reported in accordance with the Strengthening the Reporting of Observational Studies in Epidemiology (STROBE) guidelines.

We conducted a retrospective cohort study (Fig. 1) at the Affiliated Hospital of Guilin Medical University from December 2022 to July 2024, the period immediately following China’s official declaration of the end of the COVID-19 pandemic. We enrolled children aged 28 days to 5 years who were hospitalized with CAP if they presented with acute onset of lower respiratory tract symptoms (cough, sputum production, chest pain, wheeze, dyspnea, or fever) and met either of the following: (i) lung consolidation and/or moist rales on physical examination or (ii) alveolar or interstitial infiltrates on chest imaging (X-ray and/or CT). We required CAP diagnosis to be confirmed within 24 h of admission. Exclusion criteria included hospital-acquired or aspiration pneumonia; tracheobronchial foreign body; parasitic lung disease; tuberculosis; chronic pneumonia or diffuse interstitial lung disease; and lack of viral PCR and/or antigen testing within 24 h. Severe CAP (SCAP) was defined according to the 2024 Chinese guidelines as pneumonia complicated by hypoxemia requiring ventilatory support, gas-exchange impairment, or intra- or extra-pulmonary complications (Subspecialty Group of Respiratory, the Society of Pediatrics, Chinese Medical Association, Editorial Board, Chinese Journal of Pediatrics, China Medicine Education Association Committee on Pediatrics, 2024).

**Figure 1:**
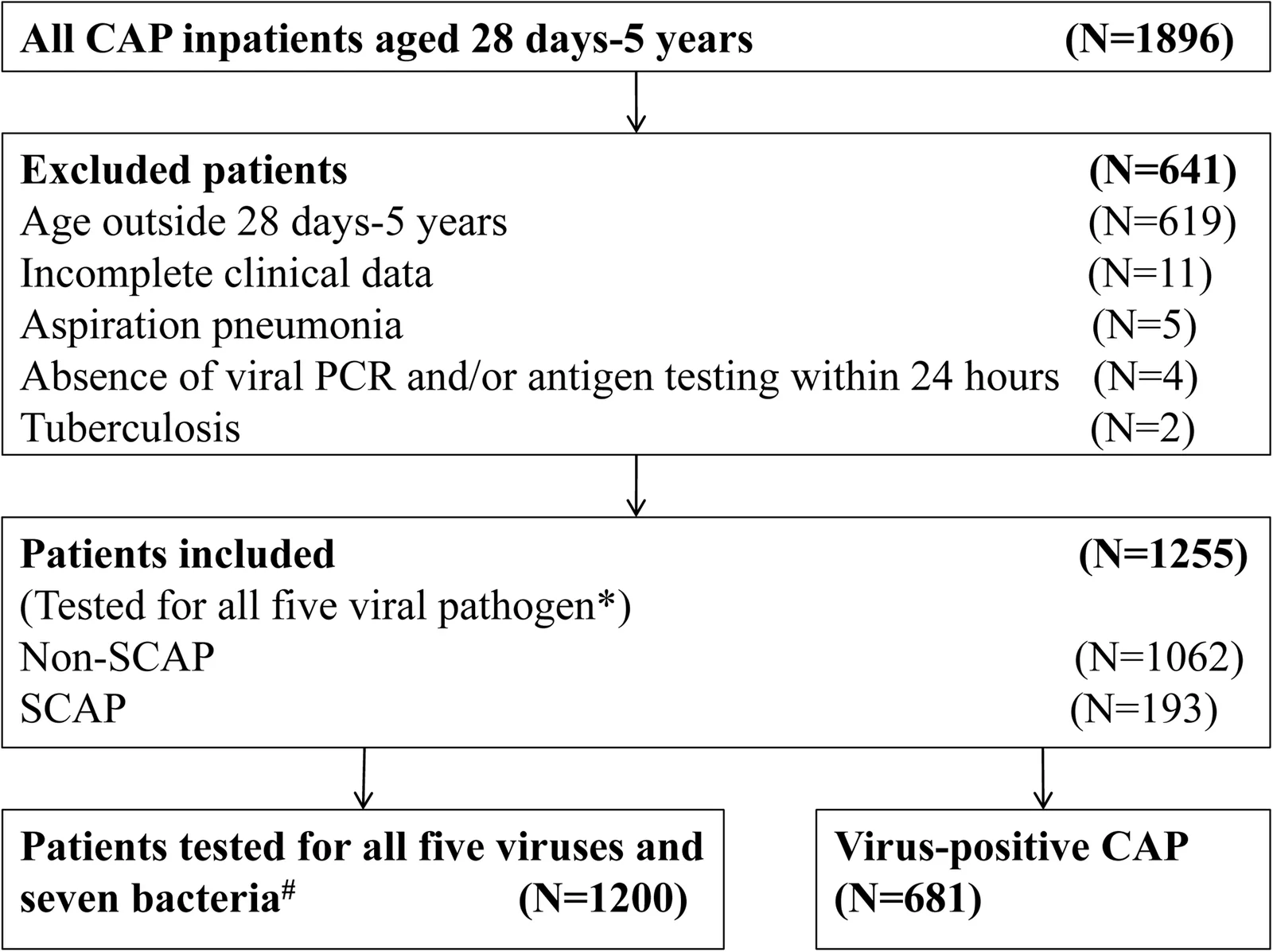
Flow chart showing enrollment of pediatric CAP inpatients (December 2022–July 2024). *Including respiratory syncytial virus, parainfluenza virus, adenovirus, influenza A and influenza B. ^#^Including Mycoplasm*a pneumoniae*, *Streptococcus pneumoniae*, *Pseudomonas aeruginosa*, *Haemophilus influenzae*, *Legionella pneumophila*, *Staphylococcus aureus* and *Klebsiella pneumoniae*. CAP, community-acquired pneumonia.

In this study, we stratified children into three age groups (≤1 year, 1–3 years, and 3–5 years) based on developmental, immunological, and behavioral differences.

### Pathogen detection

Throat swab and serum specimens were collected within 24 h of admission from all enrolled children with CAP.

Viral detection: Swabs were analyzed using a multiplex real-time Polymerase Chain Reaction (PCR) panel (Multiple Respiratory Viruses Panel; Beijing Applied Biological Technologies Co., Ltd., Beijing, China) targeting RSV, ADV, PIV, Flu-A, and Flu-B; sera were analyzed by IgM serology (Autobio Diagnostics Co., Ltd., Henan Province, China) for the five aforementioned viruses and four atypical bacteria (*Legionella pneumophila* (LP), *Mycoplasma pneumoniae* (MP), *Chlamydia pneumoniae*, and *Coxiella burnetii*). Virus-positive CAP was defined as detection of at least one of the five target pathogens (RSV, ADV, PIV, Flu-A, and Flu-B) by real-time PCR on swabs or serology on serum, and classified accordingly.

Bacterial detection: Swabs were analyzed using two PCR assays: (i) a multiplex real-time PCR panel (Multiple Respiratory Bacterial Panel; Sansure Biotech Inc., Beijing, China) targeting LP, *Streptococcus pneumoniae* (SP), *Pseudomonas aeruginosa* (PA), *Haemophilus influenzae* (HI), S*taphylococcus aureus* (SA), and *Klebsiella pneumoniae* (KP); and (ii) a duplex real-time PCR kit for MP and *Chlamydia pneumoniae* (Shanghai ZJ Bio-Tech Co., Ltd., Shanghai, China). Sera were analyzed by IgM serology for the nine respiratory pathogens listed above (Autobio Diagnostics Co., Ltd., Henan Province, China) and MP-specific antibody testing (IgM and IgG) (Autobio Diagnostics Co., Ltd., Henan, China). Bacteria-positive CAP was defined as detection of at least one of the seven routine panel pathogens (LP, SP, PA, HI, SA, KP, and MP) by real-time PCR on swabs or serology on serum, and classified accordingly.

Systematic bacterial culture of sputum or blood was not routinely performed in this study; consequently, quantitative culture results and sputum quality assessments were unavailable.

Co-detection was defined as the identification of two or more pathogens in the same patient, irrespective of specimen type.

### Data collection

Demographics (age, sex, maternal information), clinical presentation (symptoms, radiological features, and physical findings), laboratory results, and therapeutic interventions were extracted from electronic medical records. All examinations and laboratory tests were ordered on clinical grounds but performed only after guardian’s consent was obtained; thus, the diagnostic work-up varied among children.

Written informed consent for the use of de-identified medical information in future research was obtained from parents or legal guardians at the time of admission. The study protocol was reviewed and approved by the Medical Ethics Committee of the Affiliated Hospital of Guilin Medical University (No. 2025IITLL-54). The Committee determined that this study fell within the scope of the original general consent and that no additional informed consent was required. All children’ data were anonymized, and no child could be identified in this retrospective study.

### Statistical analysis

All analyses were performed with SPSS version 22.0 (IBM Corp., Armonk, NY, USA). Categorical variables were described as frequencies (proportions) and positive rates; group comparisons were evaluated by χ^2^ test or Fisher’s exact test, as appropriate. Continuous variables that were not normally distributed were described as medians with interquartile ranges (IQRs); group comparisons were evaluated by the Mann-Whitney U test. Univariate analysis (*p* < 0.20) identified candidate predictors of CAP severity; these plus age—forced into the model because of its known association with virus-associated etiology—were entered into a multivariable logistic regression, and results were expressed as odds ratios (ORs) with 95% confidence intervals (CIs). All tests were two-tailed, and *p* < 0.05 was considered statistically significant.

## Results

### Cohort characteristics and viral profile in pediatric CAP

Between December 2022 and July 2024, 1,255 children younger than 5 years with CAP were enrolled in this study, 803 (64%) were male; the median age was 1.83 years (IQR 0.75–3.33) and median length of hospital stay (LOS) was 6 days (IQR 5–7) (Table S1). No in-hospital deaths occurred.

Among the 1,255 children with CAP tested for all five viral pathogens, 681 (54%) had at least one positive detection. The overall detection rate of viral pathogens in 1,255 CAP children was comparable between sexes (55% in males *vs*. 52% in females; *p* = 0.33) and showed no significant age-dependent gradient across the three age groups (*p* = 0.22): infants (age ≤ 1 year; 57% [246/434]), toddlers (age 1–3 years old; 55% [236/429]), and preschoolers (age 3–5 years old; 51% [199/392]). However, some specific viral pathogens, such as RSV and ADV, showed significant age-dependent gradient across the three pediatric strata (*p* = 2.68E−09 and *p* = 0.001, respectively). RSV had the highest overall detection rate (23% [292/1,255]), followed by PIV, ADV, Flu-A, and Flu-B. The rank order of the top three viral pathogens differed across age groups. Specifically, RSV, PIV and ADV were the three most prevalent viral pathogens in infants; the same viruses—but with ADV overtaking PIV—led in toddlers; ADV, PIV and RSV were the three leading pathogens among preschoolers. Details were shown in Table 1.

**Table 1:** Detection rate of respiratory viruses among children with CAP (*n* = 1,255) by age and gender in Guilin, China, Dec 2022–Jul 2024.

	Overall	Age group				Sex		
		Infants (≤1 y)	Toddlers (1–3 y)	Preschoolers (3–5 y)	*p* value*	Male	Female	*p* value^†^
RSV	292 (23)	140 (32)	98 (23)	54 (14)	2.68E−9	192 (24)	100 (22)	0.47
ADV	174 (14)	39 (9)	69 (16)	66 (17)	0.001	113 (14)	61 (14)	0.78
PIV	196 (16)	80 (18)	60 (14)	56 (14)	0.14	118 (15)	78 (17)	0.23
Flu-A	98 (8)	25 (6)	34 (8)	39 (10)	0.08	62 (8)	36 (8)	0.88
Flu-B	55 (4)	15 (3)	16 (4)	24 (6)	0.13	43 (5)	12 (3)	0.03
Virus^#^	681/1,255 (54)	246/434 (57)	236/429 (55)	199/392 (51)	0.22	444/803 (55)	237/452 (52)	0.33

**Notes:**

Data were *n* (%).

CAP, community-acquired pneumonia; RSV, respiratory syncytial virus; PIV, parainfluenza virus; ADV, adenovirus; Flu-A, influenza-A; Flu-B, influenza-B.

* *P* values were obtained with χ^2^ test across the three age groups (infants, toddlers, and preschoolers).

† *P* values were obtained with χ^2^ test by comparing male *vs*. female group. The pathogen detection rate was defined as the number of positive test results divided by the total number of tests performed for that pathogen. The overall viral detection rate of 1,255 CAP children who had all the five viruses tested.

# Virus: detection rate of at least one of the five viruses listed above.

Of the 1,255 CAP children, 193 (15%) met the criteria for SCAP. Among the 193 children with SCAP tested for all five viral pathogens, 112 (58%) had at least one positive detection. The overall detection rate of viral pathogens was highest in infants (64%), followed by toddlers (55%) and preschoolers (53%), with no significant difference among the three groups (*p* = 0.34). The overall rate of viral detection was significantly higher in girls than in boys (68% *vs*. 53%; *p* = 0.045); furthermore, higher viral positivity rates were observed in SCAP than in non-SCAP in girls (68% *vs*. 50%, *p* = 0.007); whereas no such difference was observed in boys (53% *vs*. 56%, *p* = 0.58). In SCAP, RSV had the highest positive detection rate (29%, 56/193), followed by ADV, PIV, Flu-A, and Flu-B. Also, the rank order of the three most prevalent viruses shifted with age: RSV, PIV and ADV in infants; RSV, ADV and PIV in toddlers; and ADV, PIV and Flu-A in preschoolers. Details were shown in Table 2.

**Table 2:** Detection rate of respiratory viruses among children with SCAP (*n* = 193) and non-SCAP (*n* = 1,063) by age group and gender in Guilin, China, Dec 2022–Jul 2024.

	Total			Age group									Sex					
				Infants (≤1 y)			Toddlers (1–3 y)			Preschoolers (3–5 y)			Male			Female		
	Non-SCAP	SCAP	*p* value	Non-SCAP	SCAP	*p* value	Non-SCAP	SCAP	*p* value	Non-SCAP	SCAP	*p* value	Non-SCAP	SCAP	*p* value	Non-SCAP	SCAP	*p* value
RSV	236 (22)	56 (29)	0.04	106 (30)	34 (45)	0.01	80 (22)	18 (27)	0.35	50 (15)	4 (7.8)	0.19	152 (23)	40 (31)	0.045	84 (22)	16 (25)	0.50
ADV	143 (13)	31 (16)	0.34	29 (8)	10 (13)	0.16	59 (16)	10 (15)	0.82	55 (16)	11 (22)	0.33	97 (14)	16 (12)	0.53	46 (12)	15 (24)	0.01
PIV	167 (16)	29 (15)	0.81	66 (18)	14 (18)	1.00	54 (15)	6 (9.1)	0.21	47 (14)	9 (17.6)	0.46	104 (15)	14 (11)	0.17	63 (16)	15 (24)	0.14
Flu-A	87 (8)	11 (6)	0.24	24 (7)	1 (1.3)	0.07	31 (9)	3 (4.5)	0.27	32 (9)	7 (13.7)	0.33	58 (9)	4 (3.1)	0.03	29 (7)	7 (11.1)	0.32
Flu-B	52 (5)	3 (1.6)	0.04	15 (4)	0 (0.0)	0.07	14 (4)	2 (3.0)	0.74	23 (7)	1 (2.0)	0.18	41 (6)	2 (1.5)	0.04	11 (3)	1 (1.6)	0.57
Total^#^	569/1,062 (54)	112/193 (58)	0.25	197/358 (55)	49/76 (64)	0.13	200/363 (55)	36/66 (55)	0.93	172/341 (50)	27/51 (53)	0.74	375/673 (56)	69/130 (53)	0.58	194/389 (50)	43/63 (68)	0.01

**Notes:**

Data were *n* (%). SCAP, severe community-acquired penumonia; RSV, respiratory syncytial virus; PIV, parainfluenza virus; ADV, adenovirus; Flu-A, influenza-A; Flu-B, influenza-B. *P* values were obtained with χ^2^ test or Fisher’s exact test by comparing the non-SCAP group *vs*. SCAP group. The pathogen detection rate was defined as the number of positive test results divided by the total number of tests performed for that pathogen. The overall viral detection rate of 193 SCAP children who had all the five viruses tested.

# Total: detection rate of at least one of the five viruses listed above.

Furthermore, 1,200 children with CAP were tested for all five viruses and seven bacteria. 653 had at least one viral positive detection; of these, 142 children (22%) had single-virus detection, 26 (4%) had viral-viral co-detections, whereas the dominant pattern was viral-bacterial co-detection, observed in 485 children (74%). The viral–bacterial co-detection increased with age (*p* = 1.80E−11), whereas viral-viral co-detection and viral single detection decreased with age (*p* = 0.014, *p* = 7.71E−9, respectively) (Table 3). The rates of single virus detection and viral co-detection were comparable between sexes (all *p* > 0.05) (Table 3). RSV single detection was most common (80/653), followed by RSV-MP co-detection (61/653), PIV-MP (41/653), PIV single detection (35/653), ADV-MP-SP triple detection (31/653), ADV-MP co-detection (23/653), Details were shown in Table S2.

**Table 3:** Viral detection and co-detection patterns in children with viral CAP (*n* = 653) by age and gender.

	Viral single detection	Viral-bacterial	Viral-viral	Co-detection
Age group, *n* (%)				
Infants (≤1 y) (*n* = 231)	81 (57)	134 (28)	16 (62)	150 (29)
Toddlers (1–3 y) (*n* = 226)	34 (24)	188 (39)	4 (15.4)	192 (38)
Preschoolers (3–5 y) (*n* = 196)	27 (19)	163 (34)	6 (23.1)	169 (33)
*P* value	7.71E−9	1.80E−11	0.01	7.71E−9
Sex, *n* (%)				
Male (*n* = 427)	96 (68)	315 (65)	16 (62)	331 (65)
Female (*n* = 226)	46 (32)	170 (35)	10 (38)	180 (35)
*P* value	0.53	0.69	0.67	0.53
Total	142/653 (22)	485/653 (74)	26/653 (4.0)	511/653 (78)

**Note:**

Data were *n* (%).

CAP, community-acquired pneumonia. The χ^2^ test was used for comparisons among age groups and gender groups. Among 1,200 CAP children tested for all 12 pathogens, 653 had ≥1 virus detected; the frequency and proportion of viral single detection and co-detection (viral-bacterial or viral-viral) were calculated within the viral-positive subset.

### Clinical characteristics and risk factors for severe virus-positive CAP

Among the 681 children with virus-positive CAP, 444 (65%) were male; the median age was 1.67 years (IQR 0.71–3.25) and median LOS was 6.00 days (IQR 5.00–7.00). Of the 681 virus-positive CAP children, 112 (16%) met the criteria for SCAP. Compared with non-SCAP, children with SCAP had a longer LOS (median [IQR]: 7.00 [6.00–9.00] *vs*. 6.00 [5.00–7.00]; Table 4).

**Table 4:** Demographic, obstetric, clinical, and microbiological characteristics of children with viral CAP in Guilin, China, Dec 2022–Jul 2024 (*n* = 681).

Demographic	Total (*n* = 681)	Non-SCAP (*n* = 569)	SCAP (*n* = 112)	*p* value*
Age, median (IQR)	1.67 (0.71–3.25)	1.75 (0.75–3.25)	1.29 (0.58–2.98)	0.08
Age group	–	–	–	0.17
≤1 y (*n*, %)	246 (36)	197 (35)	49 (44)	–
1–3 y (*n*, %)	236 (35)	200 (35)	36 (32)	–
3–5 y (*n*, %)	199 (29)	172 (30)	27 (24)	–
Gender	–	–	–	0.38
Male (*n*, %)	444 (65)	375 (66)	69 (62)	–
Female (*n*, %)	237 (35)	194 (34)	43 (38)	–
LOS, median (IQR)	6.00 (5.00–7.00)	6.00 (5.00–7.00)	7.00 (6.00–9.00)	3.84E−14
Obstetric characteristic				
Twin gestation	–	–	–	0.49
Yes (*n*, %)	15 (2)	14 (2)	1 (0.9)	–
No (*n*, %)	666 (98)	555 (98)	111 (99)	–
Preterm birth	–	–	–	0.77
Yes (*n*, %)	62 (9)	51 (9)	11 (10)	–
No (*n*, %)	619 (91)	518 (91)	101 (90)	–
Cesarean delivery	–	–	–	0.21
Yes (*n*, %)	217 (32)	187 (33)	30 (27)	–
No (*n*, %)	464 (68)	382 (67)	82 (73)	–
Clinical features				
Cough (*n*, %)	668 (98)	558 (98)	110 (98)	1.00
Expectoration (*n*, %)	484 (71)	408 (72)	76 (68)	0.41
Rhonchi (*n*, %)	81 (12)	66 (12)	15 (13)	0.59
Stuffy nose (*n*, %)	179 (26)	152 (27)	27 (24)	0.57
Sneeze (*n*, %)	8 (1.2)	7 (1.2)	1 (0.9)	1.00
Rhinorrhoea (*n*, %)	333 (49)	295 (52)	38 (34)	0.001
Headache (*n*, %)	11 (2)	10 (2)	1 (0.9)	1.00
Fever (*n*, %)	526 (77)	446 (78)	80 (71)	0.11
Vomit (*n*, %)	133 (20)	111 (20)	22 (20)	0.97
Wheeze (*n*, %)	209 (31)	163 (29)	46 (41)	0.01
Tachypnea (*n*, %)	76 (11)	32 (6)	44 (39)	4.56E−25
Bellyache (*n*, %)	23 (3)	21 (4)	2 (1.8)	0.40
Diarrhea (*n*, %)	26 (4)	21 (4)	5 (4.5)	0.60
Altered mental status^⸸^ (*n*, %)	192 (28)	135 (24)	57 (51)	5.19E−9
Sleep disturbance (*n*, %)	177 (26)	142 (25)	35 (31)	0.17
Poor appetite (*n*, %)	358 (53)	291 (51)	67 (60)	0.09
Chill (*n*, %)	36 (5)	31 (5)	5 (4.5)	0.67
Shiver (*n*, %)	35 (5)	29 (5)	6 (5.4)	0.91
Twitch (*n*,%)	11 (2)	10 (2)	1 (0.9)	1.00
Rash (*n*, %)	12 (2)	11 (2)	1 (0.9)	0.70
Stridor (*n*, %)	71 (10)	60 (11)	11 (10)	0.82
Comorbid chronic disease(s)^#^	202 (30)	151 (27)	51 (46)	5.70E−5
Antibiotic treatment	676 (99)	564 (99)	112 (100)	1.00
Viral pathogens				
RSV	292 (43)	236 (41)	56 (50)	0.10
ADV	174 (26)	143 (25)	31 (28)	0.57
PIV	196 (29)	167 (29)	29 (26)	0.46
Flu-A	98 (14)	87 (15)	11 (10)	0.13
Flu-B	55 (8)	52 (9)	3 (2.7)	0.02
Bacterial co-detection	–	–	–	0.13
Yes (*n*, %)	495 (73)	407 (72)	88 (79)	–
No (*n*, %)	186 (27)	162 (28)	24 (21)	–

**Notes:**

Data were *n* (%). CAP, community-acquired pneumonia; SCAP, severe community-acquired pneumonia; IQRs, interquartile ranges; LOS, length of hospital stay; RSV, respiratory syncytial virus; ADV, adenovirus; PIV, parainfluenza virus; Flu-A, influenza virus-A; Flu-B, influenza virus-B.

⸸ Altered mental status was defined as agitation, lethargy, or unconsciousness observed during the acute phase of CAP relative to the child’s baseline status, as assessed by parents and physicians.

* *P* values were obtained with Mann-Whitney U test (continuous variables), or χ^2^ test or Fisher’s exact test (categorical variables) by comparing the non-SCAP group *vs*. SCAP group.

# Comorbid chronic disease(s) including asthma, bronchopulmonary dysplasia, epilepsy, leukemia, immune deficiency, hyperthyroidism, birth defects (*e.g*., congenital heart diseases, congenital genetic metabolic diseases, renal malformations, cerebral ventricular malformations) and other systems involved chronic conditions.

While baseline obstetric and demographic characteristics (gender, age distribution, twin gestation, preterm birth and cesarean delivery) were comparable between the SCAP and non-SCAP groups (all *p* > 0.05; Table 4), clinical features including rhinorrhoea, wheeze, tachypnoea, altered mental status, and comorbid chronic disease were significantly associated with SCAP (all *p* < 0.05; Table 4), Notably, Flu-B was the only viral pathogen significantly linked to SCAP (*p* = 0.022; Table 4).

To identify independent determinants of severity among children with virus-positive CAP, variables with *p* < 0.20 in univariate analyses were subsequently entered into a multivariable binary logistic regression model. Tachypnea and altered mental status were excluded to avoid circularity in severity assessment; Flu-B was additionally excluded due to limited SCAP cases (*n* = 3). After multivariable adjustment, bacterial co-detection (OR 1.90, 95% CI [1.12–3.23]), wheeze (OR 1.59, 95% CI [1.02–2.49]) and chronic comorbidities (OR 2.44, 95% CI [1.57–3.78]) were independently associated with increased disease severity, whereas rhinorrhoea (OR 0.43, 95% CI [0.27–0.66]) was associated with reduced risk (Fig. 2).

**Figure 2:**
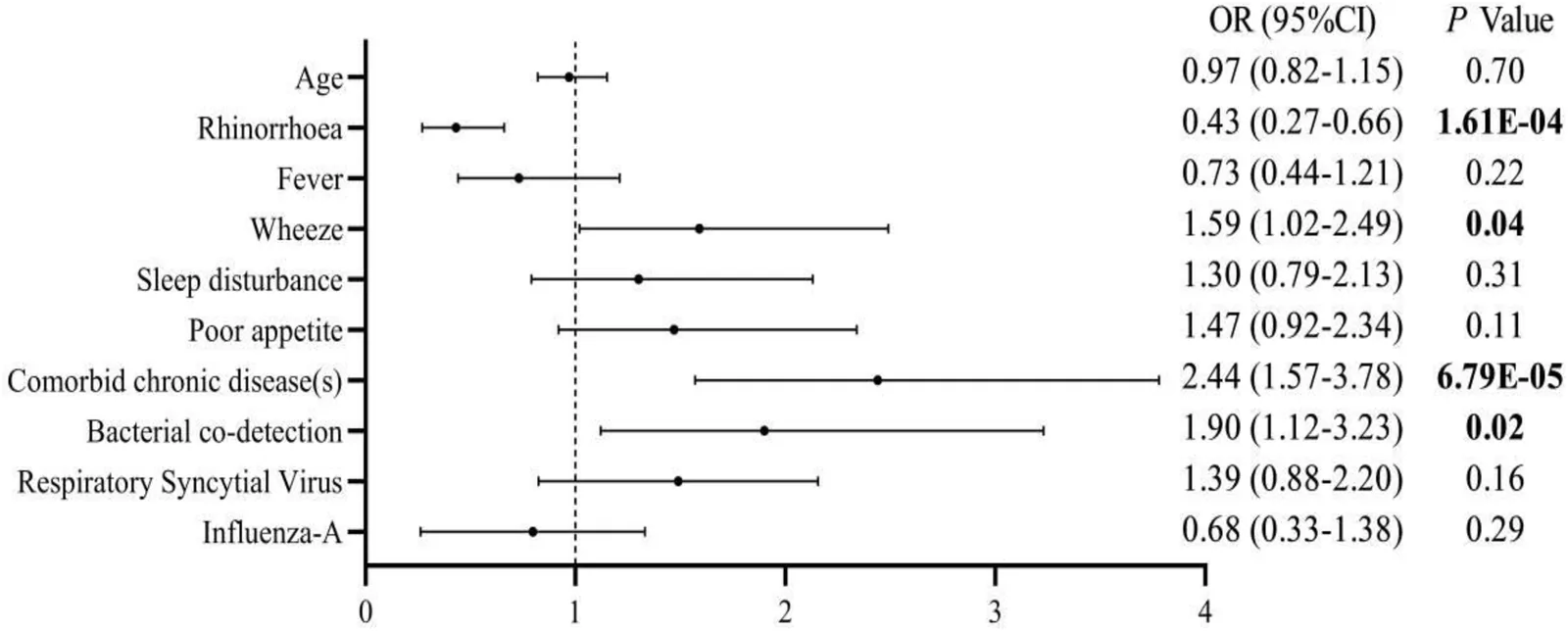
Identification of independent risk factors for virus-positive CAP severity by logistic regression model. Model diagnostics showed no multicollinearity (VIF range 1.02–1.25, tolerance > 0.80, condition index = 9.75); Calibration was adequate (Hosmer-Lemeshow test: χ^2^ = 10.94, df = 8, *p* = 0.205); Discrimination was acceptable (C-statistic 0.70, 95% CI [0.65–0.76], *p* < 0.001); Goodness-of-fit: Nagelkerke R^2^ = 0.12. Bold values in the *P* value column indicate statistical significance (*P* < 0.05); these variables were identified as independent risk factors for virus-positive CAP severity. 95% CI, 95% confidence interval; OR, odds ratio; CAP, community-acquired pneumonia.

### Temporal dynamics of the five common respiratory viruses after NPIs relaxation (December 2022–July 2024)

After the withdrawal of NPIs in November 2022, we tracked monthly case counts and positivity rates of five respiratory viruses among children with CAP in Guilin from December 2022 to July 2024 to delineate post-NPI epidemiological shifts (Fig. 3). Data collection concluded on 31 July 2024, thereby precluding assessment trends in the second half of 2024.

**Figure 3:**
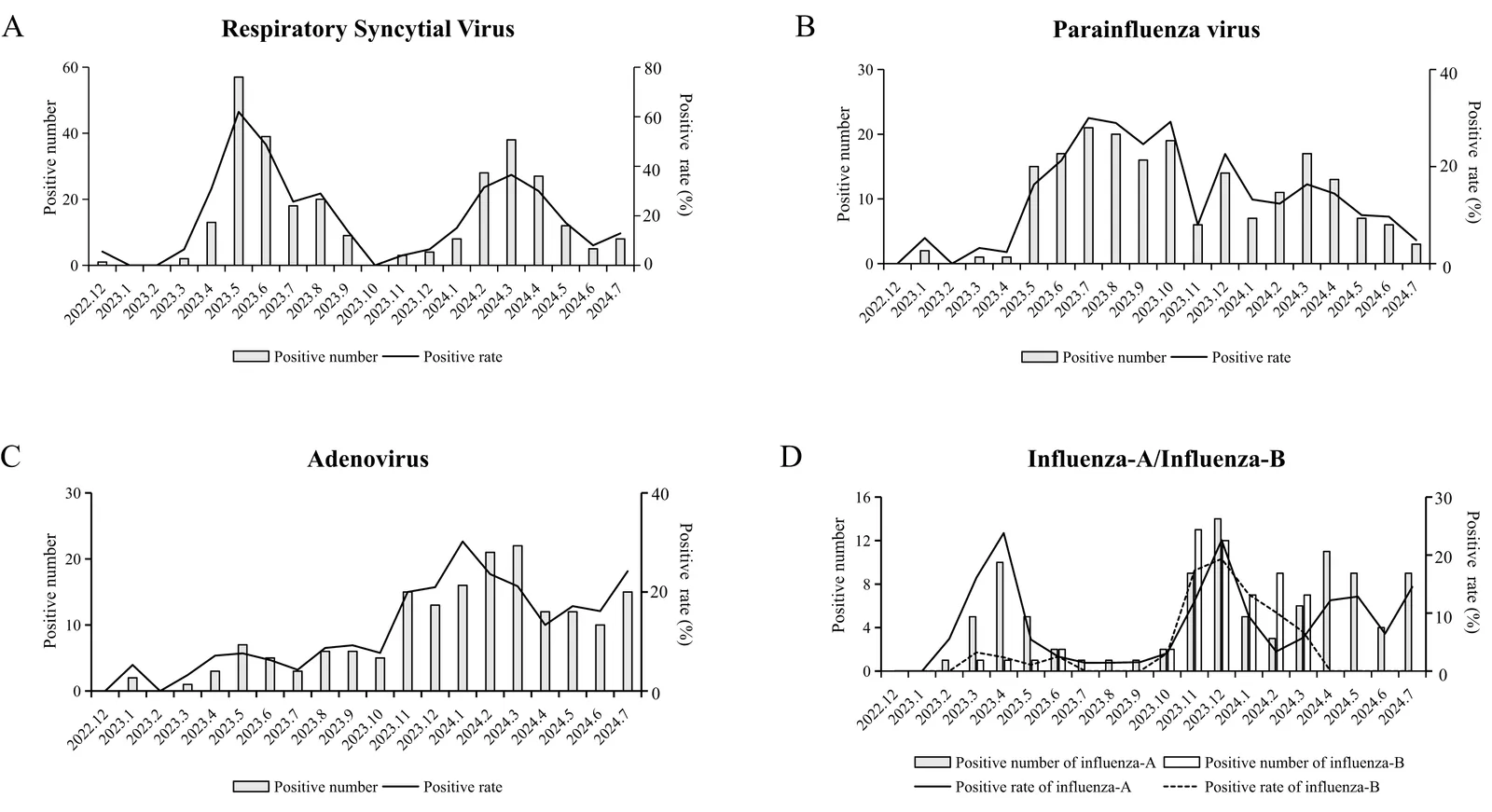
Temporal dynamics of the five common respiratory viruses after relaxation of non-pharmaceutical interventions (NPIs) (December 2022–July 2024). (A) Respiratory syncytial virus; (B) Parainfluenza virus; (C) Adenovirus; (D) Influenza A and influenza B. Monthly positivity rate was calculated as the proportion of CAP patients testing positive for the target pathogen among all CAP patients during that month.

RSV exhibited marked deviation from its typical winter-season pattern: positivity remained negligible from December 2022 through February 2023 (0% in January–February 2023) before surging to a delayed spring peak of 62% (57 cases) in May 2023. Subsequently, rates declined progressively, stabilizing below 30% after July 2023. A second, smaller peak (37%, 38 cases) emerged in March 2024, indicating inter-seasonal recirculation. Notably, no winter epidemic was observed in either 2022–2023 or 2023–2024, underscoring the marked inter-annual shift in epidemic timing. Details were shown in Fig. 3A.

PIV exhibited a multi-peaked distribution lacking a distinct primary maximum: following similarly suppressed activity from December 2022 to April 2023 (0–2 cases monthly; 0–5.3%), positivity surged to 30% (21 cases) in July 2023, forming a summer-predominant epidemic that remained above 20% for five consecutive months (June–October 2023). A second, lower peak (23%) emerged in December 2023, followed by a third, attenuated wave peaking at 16% in March 2024. Details were shown in Fig. 3B.

ADV circulated at low levels for nearly one year after NPIs withdrawal (December 2022–October 2023: 0–7 cases monthly; 0–7.7%), then climbed to approximately 20% positivity in November–December 2023. In 2024, ADV activity intensified relative to the corresponding period in 2023, with positivity rates ranging from 13 to 30%. Details were shown in Fig. 3C.

Flu-A circulated at low levels (positive rate: 0–5.6%) during the canonical winter season (December 2022–February 2023), followed by a spring surge from 16% in March to a study-period maximum of 24% in April 2023, subsequently declining to ≤6% during May–October 2023. A winter resurgence (12–23%) appeared in November–December 2023, and an attenuated third peak (13%) emerged in May 2024. Thereafter, rates stabilized at 6–14% through July 2024, indicating sustained low–level endemicity rather than complete inter-epidemic silence. Details were shown in Fig. 3D.

Flu-B showed only a transient winter-spring wave (November 2023–March 2024; peak 19%, 12 cases) superimposed on a background of persistently low activity throughout the study period. Details were shown in Fig. 3D.

## Discussion

The epidemiology of CAP in young children varies considerably across regions, reflecting differences in pathogen distribution, demographic characteristics, and local risk factor profiles (DeAntonio et al., 2016; Roh, Shim & Chung, 2022; Rudan et al., 2008). In this study, conducted after the withdrawal of COVID-19 NPIs, we identified virus-positive CAP in 54% (681/1,255) of all CAP cases among children younger than 5 years in Guilin from December 2022 to July 2024. RSV was the predominant viral pathogen. Of these 681 virus-positive children, 16% (112/681) met the criteria for SCAP.

Early recognition of SCAP improves outcomes by reducing mortality (Franklin et al., 2023), shortening hospital stay, minimizing long-term sequelae (Ralston et al., 2014), and conserving scarce resources. In this cohort, chronic comorbidities, bacterial co-detection, and wheeze were independently associated with severe virus-positive CAP, whereas rhinorrhoea was associated with reduced risk. These findings have direct implications for risk stratification, clinical management, and health resource allocation in children hospitalized with virus-positive CAP. Chronic diseases such as congenital heart disease (CHD), bronchopulmonary dysplasia (BPD) and asthma confer anatomical and immunological vulnerability, promoting pneumonia deterioration (Collaco & McGrath-Morrow, 2024; Desai et al., 2022; Torres et al., 2015). Consequently, when managing children with CAP, clinicians should obtain detailed histories of underlying chronic diseases to facilitate early risk stratification and lower thresholds for escalation of care. Viral-bacterial co-infection can synergistically disrupt the airway epithelial barrier, enhance susceptibility to invasive pathogens, and amplify inflammatory responses, thereby increasing mortality risks; consequently, it has been proposed as an independent risk factor of SCAP (Klein et al., 2016; McCullers, 2006). In contrast, Waldeck et al. (2025) observed no significant differences in clinical presentation, LOS, CAP-related complications, or duration of antibiotic therapy across four groups (bacteria-only, virus-only, virus–bacteria co-infection, and no pathogen detected). These seemingly discrepant results most likely reflect heterogeneity in sample size, pathogen composition, host factors, and study design. In this study, we identified bacterial co-detection as an independent risk factor for virus-positive SCAP, suggesting that children with confirmed or suspected viral-bacterial co-detection warrant closer monitoring, prompt antimicrobial therapy, and lower thresholds for escalation of care. Wheeze has been suggested as a potential predictor of viral infection (Nascimento-Carvalho, Ruuskanen & Nascimento-Carvalho, 2019) and reflects airway hyperreactivity (Chen et al., 2021). It has also been identified as an independent risk factor for SCAP in children (Chen et al., 2021). Clinicians should therefore maintain heightened vigilance for airway obstruction progression in wheezing children with CAP. Finally, rhinorrhoea was associated with reduced SCAP risk, possibly reflecting enhanced upper-airway mucociliary clearance and early viral removal (Hariri & Cohen, 2016). Thus, integrating these risk signals into bedside decision-making can halt progression to severe disease, enhance the efficiency of supportive and targeted therapy, and ultimately reduce virus-positive CAP burden.

RSV is the most frequently detected respiratory pathogen in children with CAP, accounting for 7–48% of cases (Pratt et al., 2022). Consistently, we identified RSV as the dominant viral pathogen in young children with virus-positive CAP in Guilin and as the most common virus in co-detections. Recent studies report RSV infection as an independent determinant of SCAP (Bardsley et al., 2023; Ren et al., 2023), particularly in children younger than 5 years (Haddadin et al., 2021). In contrast, our multivariable regression analysis showed no independent association between RSV and SCAP, a finding corroborated by other research groups (Kim et al., 2019; Waldeck et al., 2025). Such discrepancies likely reflect differences in sample size, geographic setting, patient age, comorbidity burden, and immune status, rather than RSV itself.

Notably, RSV exhibited classic winter seasonality in pediatric CAP before the COVID-19 pandemic (Feng et al., 2025; Rose et al., 2018). However, pandemic-era NPIs substantially suppressed RSV circulation and markedly reduced detection rates. Following the withdrawal of NPIs, RSV re-emerged with an atypical spring-summer peak. This temporal shift occurred globally: in Australia and South Africa, RSV activity peaked in spring and summer 2020, coinciding with the implementation and subsequent easing of NPIs; in the United States, RSV peaked in spring and summer 2021, with geographical heterogeneity linking to varying NPI stringency (Chow, Uyeki & Chu, 2023; Li et al., 2024; Xu, Wu & Liu, 2025). Similarly, we observed an off-season surge of RSV in Guilin during the spring and summer of 2023. Potential factors include: (1) the accumulation of susceptible individuals during prolonged NPIs, resulting in reduced natural exposure and/or declining maternal antibody levels (Chow, Uyeki & Chu, 2023; Li et al., 2024); (2) increased social mixing among children following the lifting of NPIs; (3) heightened health-seeking behavior by caregivers in the post-pandemic era; (4) expanded respiratory virus testing; and (5) Guilin’s subtropical monsoon climate, where RSV activity correlates with elevated humidity during the rainy spring–summer period. These data underscore the need for year-round molecular surveillance of RSV. Characterizing the epidemiological patterns of RSV and other respiratory viruses helps guide effective prevention and control strategies.

Additionally, the high rate of antibiotic use (99%, 676/681) observed in this cohort (Table 4), despite the absence of bacterial pathogens in 27% of cases, highlights a fundamental tension in antimicrobial stewardship. The inability to rapidly distinguish bacterial from viral etiologies at admission, coupled with the severity of illness in hospitalized children, often leads to empirical broad-spectrum coverage to prevent missed bacterial infections (Kolberg et al., 2024). However, this practice contributes to antibiotic overuse, potentially accelerating selection for resistance and increasing healthcare costs (Ventola, 2015). Future integration of rapid diagnostics (*e.g*., multiplex PCR, procalcitonin) may guide antibiotic de-escalation in viral-dominant CAP, and align empirical therapy with stewardship principles (Lee et al., 2020).

This study had several limitations. First, the single pediatric center design may limit generalizability. Second, the absence of pre-pandemic and pandemic-period comparison data precludes assessment of COVID-19 impacts on viral circulation. Third, the lack of quantitative disease-severity data precluded assessment of outcome with antiviral or adjunctive therapies (*e.g*., immunomodulators, traditional Chinese medicine). Fourth, reliance on routine clinical diagnostics precluded assessment of other CAP-associated pathogens (*e.g*., rhinovirus, SARS-CoV-2, *Group A Streptococcus*, and *Moraxella catarrhalis*), potentially compromising pathogen detection completeness. Fifth, retrospective reliance on upper respiratory PCR and single time point serology—without quantitative bacterial culture or paired sera—precluded definitive discrimination between infection, colonization, and bystander presence, potentially leading to overestimation of true infection and co-infection rates. Sixth, vaccination histories were unavailable due to lack of integration between hospital electronic medical records and community immunization registries in China, precluding stratification by vaccine status.

## Conclusions

In summary, we characterized the epidemiologic and clinical profile of post-NPI virus-positive CAP in Guilin among children younger than 5 years and identified independent risk and protective factors for disease severity. These findings inform prevention, treatment, and healthcare resource allocation for virus-positive CAP in this region and in comparable resource-limited settings.

## Supplemental Information

10.7717/peerj.21849/supp-1 Supplemental Information 1 Raw data for all children.

10.7717/peerj.21849/supp-2 Supplemental Information 2 Raw data for Fig 2.

10.7717/peerj.21849/supp-3 Supplemental Information 3 Raw data for Fig 3.

10.7717/peerj.21849/supp-4 Supplemental Information 4 Demographic characteristics of children with CAP in Guilin, China, Dec 2022 – Jul 2024 (n = 1255). CAP: community-acquired pneumonia. SCAP: severe community-acquired pneumonia. IQRs: interquartile ranges. LOS: length of hospital stay. *P values were obtained with Mann-Whitney U test (continuous variables) or χ² test (categorical variables) by comparing the non-SCAP group *vs* SCAP group.

10.7717/peerj.21849/supp-5 Supplemental Information 5 Detailed viral single detection and co-detection pattern in children with viral CAP (n=653). Data were shown as number. Among 1,200 CAP children tested for all 12 pathogens, 653 had ≥ 1 virus detected; the number of viral single detection and co-detection (viral-bacterial or viral-viral) were calculated within the viral-positive subset. Virus: RSV: respiratory syncytial virus; PIV: parainfluenza virus; ADV: adenovirus; Flu-A: influenza-A; Flu-B: influenza-B. Bacteria: MP: *Mycoplasma pneumoniae*; SP: *Streptococcus pneumoniae*; PA: *Pseudomonas aeruginosa*; KI: *Haemophilus influenzae*; LP: *Legionella pneumophila*; SA: *Staphylococcus aureus*; KP: *Klebsiella pneumoniae*.

## Additional Information and Declarations

### Data Availability

The following information was supplied regarding data availability:

The data is available in the Supplemental Files.
