# Supplementary material for: Respiratory viral detection and severity risk factors in hospitalized children with community-acquired pneumonia following relaxation of COVID-19 non-pharmaceutical interventions: a 20-month retrospective cohort in Guilin, China
Source: PeerJ. 2026 Nov 9;14:e21849. doi: 10.7717/peerj.21849 (PMC13757737; doi:10.7717/peerj.21849)
Supplement: Supplementary file 1 [file peerj-14-21849-s004.docx]

Supplementary Table 1. Demographic characteristics of children with CAP in Guilin, China, Dec 2022–Jul 2024 (n=1255).

| Demographic | Total  (n=1255) | Non-SCAP (n=1062) | SCAP  (n=193) | | *P* Value* |
| --- | --- | --- | --- | --- | --- |
| Age, median (IQR) | 1.83 (0.75-3.33) | 1.83 (0.75-3.33) | | 1.58 (0.67-3.17) | 0.19 |
| Age group | - | - | | - | 0.20 |
| ≤1y (n,%) | 434 (35) | 358 (34) | | 76 (39) | - |
| 1-3y (n,%) | 429 (34) | 363 (34) | | 66 (34) | - |
| 3-5y (n,%) | 392 (31) | 341 (32) | | 51 (26) | - |
| **Gender** | - | - | | - | 0.29 |
| Male (n,%) | 803 (64) | 673 (63) | | 130 (67) | - |
| Female (n,%) | 452 (36) | 389 (37) | | 63 (33) | - |
| **LOS, median (IQR)** | 6.00 (5.00-7.00) | 6.00 (4.00-7.00) | | 7.00 (6.00-9.00) | **1.65E-21** |

CAP: community-acquired pneumonia.

SCAP: severe community-acquired pneumonia.

IQRs: interquartile ranges.

LOS: length of hospital stay.

**P* values were obtained with Mann-Whitney U test (continuous variables) or χ² test (categorical variables) by comparing the non-SCAP group vs SCAP group.
