# Supplementary material for: Respiratory viral detection and severity risk factors in hospitalized children with community-acquired pneumonia following relaxation of COVID-19 non-pharmaceutical interventions: a 20-month retrospective cohort in Guilin, China
Source: PeerJ. 2026 Nov 9;14:e21849. doi: 10.7717/peerj.21849 (PMC13757737; doi:10.7717/peerj.21849)
Supplement: Supplementary file 2 [file peerj-14-21849-s005.docx]

Supplementary Table 2. Detailed viral single detection and co-detection pattern in children with viral CAP (n=653).

| Viral-bacterial | n | Viral-viral | n | Viral single detection | n |
| --- | --- | --- | --- | --- | --- |
| RSV+MP | 61 | RSV+PIV | 9 | RSV | 80 |
| PIV+MP | 41 | RSV+ADV | 5 | PIV | 35 |
| ADV+MP+SP | 31 | RSV+ADV+PIV | 3 | Flu-A | 14 |
| ADV+MP | 23 | PIV+Flu-A | 2 | ADV | 9 |
| RSV+MP+SP+HI | 16 | RSV+Flu-B | 1 | Flu-B | 4 |
| PIV+MP+SP | 14 | RSV+Flu-A | 1 |  |  |
| ADV+MP+SP+HI | 14 | PIV+Flu-B | 1 |  |  |
| RSV+PIV+MP | 13 | ADV+PIV | 1 |  |  |
| Flu-A+MP | 13 | ADV+Flu-A | 1 |  |  |
| ADV+SP | 12 | RSV+PIV+Flu-A | 1 |  |  |
| RSV+MP+SP | 12 | RSV+ADV+Flu-A | 1 |  |  |
| Flu-A+MP+SP | 12 |  |  |  |  |
| RSV+HI | 11 |  |  |  |  |
| PIV+MP+SP+HI | 9 |  |  |  |  |
| RSV+SP | 8 |  |  |  |  |
| PIV+SP | 8 |  |  |  |  |
| Flu-A+SP | 8 |  |  |  |  |
| RSV+SP+HI | 8 |  |  |  |  |
| Flu-A+MP+SP+HI | 8 |  |  |  |  |
| ADV+MP+HI | 7 |  |  |  |  |
| PIV+ADV+MP | 7 |  |  |  |  |
| Flu-A+SP+HI | 6 |  |  |  |  |
| ADV+SP+HI | 6 |  |  |  |  |
| Flu-B+MP+SP+HI | 6 |  |  |  |  |
| RSV+ADV+MP+SP+HI | 6 |  |  |  |  |
| Flu-B+MP+SP | 5 |  |  |  |  |
| Flu-A+MP+HI | 5 |  |  |  |  |
| Flu-B+MP | 4 |  |  |  |  |
| Flu-B+HI | 4 |  |  |  |  |
| Flu-B+SP+HI | 4 |  |  |  |  |
| Flu-B+RSV+MP | 4 |  |  |  |  |
| ADV+RSV+MP | 4 |  |  |  |  |
| Flu-B+MP+HI | 4 |  |  |  |  |
| PIV+ADV+MP+SP+HI | 4 |  |  |  |  |
| ADV+HI | 3 |  |  |  |  |
| RSV+ADV+SP | 3 |  |  |  |  |
| PIV+MP+HI | 3 |  |  |  |  |
| RSV+ADV+SP+HI | 3 |  |  |  |  |
| RSV+PIV+MP+SP | 3 |  |  |  |  |
| RSV+ADV+MP+SP | 3 |  |  |  |  |
| PIV+ADV+MP+SP | 3 |  |  |  |  |
| PIV+ADV+Flu-B+MP | 3 |  |  |  |  |
| PIV+HI | 2 |  |  |  |  |
| Flu-A+HI | 2 |  |  |  |  |
| RSV+PIV+SP | 2 |  |  |  |  |
| ADV+KP+SP | 2 |  |  |  |  |
| PIV+Flu-A+MP | 2 |  |  |  |  |
| ADV+KP+MP | 2 |  |  |  |  |
| RSV+MP+HI | 2 |  |  |  |  |
| RSV+Flu-B+HI | 2 |  |  |  |  |
| RSV+ADV+HI | 2 |  |  |  |  |
| PIV+Flu-B+MP+SP | 2 |  |  |  |  |
| ADV+Flu-A+MP+SP+HI | 2 |  |  |  |  |
| Flu-B+SP | 1 |  |  |  |  |
| PIV+PA | 1 |  |  |  |  |
| RSV+KP | 1 |  |  |  |  |
| PIV+KP | 1 |  |  |  |  |
| RSV+Flu-B+SP | 1 |  |  |  |  |
| PIV+Flu-A+SP | 1 |  |  |  |  |
| PIV+KP+SP | 1 |  |  |  |  |
| PIV+SP+HI | 1 |  |  |  |  |
| PIV+ADV+SP | 1 |  |  |  |  |
| Flu-A+ADV+SP | 1 |  |  |  |  |
| Flu-A+RSV+MP | 1 |  |  |  |  |
| RSV+PA+MP | 1 |  |  |  |  |
| PIV+LP+MP | 1 |  |  |  |  |
| Flu-A+Flu-B+MP | 1 |  |  |  |  |
| RSV+PIV+HI | 1 |  |  |  |  |
| PIV+Flu-B+HI | 1 |  |  |  |  |
| RSV+KP+HI | 1 |  |  |  |  |
| PIV+KP+HI | 1 |  |  |  |  |
| PIV+ADV+HI | 1 |  |  |  |  |
| RSV+PIV+SP+HI | 1 |  |  |  |  |
| PIV+Flu-A+SP+HI | 1 |  |  |  |  |
| RSV+PA+SP+HI | 1 |  |  |  |  |
| PIV+KP+SP+HI | 1 |  |  |  |  |
| Flu-A+KP+SP+HI | 1 |  |  |  |  |
| PIV+ADV+SP+HI | 1 |  |  |  |  |
| Flu-A+ADV+SP+HI | 1 |  |  |  |  |
| RSV+Flu-A+MP+SP | 1 |  |  |  |  |
| ADV+KP+MP+SP | 1 |  |  |  |  |
| Flu-A+Flu-B+MP+SP | 1 |  |  |  |  |
| Flu-B+ADV+MP+SP | 1 |  |  |  |  |
| Flu-A+ADV+MP+SP | 1 |  |  |  |  |
| PIV+ADV+RSV+MP | 1 |  |  |  |  |
| RSV+Flu-B+MP+HI | 1 |  |  |  |  |
| PIV+ADV+Flu-B+MP | 1 |  |  |  |  |
| PIV+ADV+PA+MP+SP | 1 |  |  |  |  |
| PIV+Flu-A+MP+SP+HI | 1 |  |  |  |  |
| Flu-A+KP+MP+SP+HI | 1 |  |  |  |  |
| Flu-A+Flu-B+MP+SP+HI | 1 |  |  |  |  |
| PIV+ADV+Flu-A+MP+SP | 1 |  |  |  |  |
| Flu-A+Flu-B+ADV+MP+SP | 1 |  |  |  |  |
| RSV+ADV+Flu-A+MP+SP+HI | 1 |  |  |  |  |

Data were shown as number.

Among 1,200 CAP children tested for all 12 pathogens, 653 had ≥1 virus detected; the number of viral single detection and co-detection (viral-bacterial or viral-viral) were calculated within the viral-positive subset.

Virus: RSV: respiratory syncytial virus; PIV: parainﬂuenza virus; ADV: adenovirus; Flu-A: influenza-A; Flu-B: influenza-B.

Bacteria: MP: Mycoplasma pneumoniae; SP: Streptococcus pneumoniae; PA: Pseudomonas aeruginosa; KI: Haemophilus influenzae; LP: Legionella pneumophila; SA: Staphylococcus aureus; KP: Klebsiella pneumoniae.
